# Structural elements in the flexible tail of the co-chaperone p23 coordinate client binding and progression of the Hsp90 chaperone cycle

**DOI:** 10.1038/s41467-021-21063-0

**Published:** 2021-02-05

**Authors:** Maximilian M. Biebl, Abraham Lopez, Alexandra Rehn, Lee Freiburger, Jannis Lawatscheck, Birgit Blank, Michael Sattler, Johannes Buchner

**Affiliations:** 1 Department of Chemistry, Technische Universität München, Garching, Germany; 2 Institute of Structural Biology, Helmholtz Zentrum München, Neuherberg, Germany; 3 Present Address: Institut für Mikrobiologie der Bundeswehr, München, Germany; 4 Present Address: Zymeworks Inc., Vancouver, BC Canada; 5 Present Address: C5 Department of Cell and Molecular Biology, CMB Farnebo, Stockholm, Sweden

**Keywords:** Chaperones, Solution-state NMR, Molecular conformation

## Abstract

The co-chaperone p23 is a central part of the Hsp90 machinery. It stabilizes the closed conformation of Hsp90, inhibits its ATPase and is important for client maturation. Yet, how this is achieved has remained enigmatic. Here, we show that a tryptophan residue in the proximal region of the tail decelerates the ATPase by allosterically switching the conformation of the catalytic loop in Hsp90. We further show by NMR spectroscopy that the tail interacts with the Hsp90 client binding site via a conserved helix. This helical motif in the p23 tail also binds to the client protein glucocorticoid receptor (GR) in the free and Hsp90-bound form. In vivo experiments confirm the physiological importance of ATPase modulation and the role of the evolutionary conserved helical motif for GR activation in the cellular context.

## Abstract (web summary)

p23 is a co-chaperone of Hsp90 but its mode of action is mechanistically not well understood. Here, the authors combine in vitro and yeast in vivo assays, biochemical measurements and NMR experiments to characterize p23 and identify two conserved helical elements in the intrinsically disordered C-terminal tail of p23 that together with the folded domain of p23 regulate the Hsp90 ATPase activity and affect the binding and maturation of Hsp90 clients.

## Introduction

The molecular chaperone p23 is an important component of the Hsp90 chaperone machinery. In contrast to many other co-chaperones it seems to be part of the core chaperone cycle required for efficient client activation^1,2^. p23 is a highly conserved protein expressed in eukaryotes ranging from yeast to man^3–6^, while prokaryotes lack orthologues. An additional, muscle-specific isoform—Aarsd1—has been identified, which replaces p23 during muscle differentiation^7^. p23 is not essential in budding and fission yeast, but it is required for perinatal survival in mice^8–10^. At least in part, the positive regulatory effect of p23 on the glucocorticoid receptor (GR) seems to be responsible for this function^8,11^.

p23 consists of two modules, a folded domain and a long unstructured C-terminal tail. The folded CHORD and Sgt1 (CS) domains of human and yeast p23 exhibit a highly similar antiparallel β-sandwich structure^12,13^ which corresponds to the fold of the α-crystallin domain of small Hsps^14^. The C-terminal extension of p23 is variable in sequence and length; it spans 56 amino acids in human p23 and 93 amino acids in yeast p23 and is largely unstructured as shown by CD spectroscopy^15,16^.

Originally, p23 was discovered as a component of the progesterone receptor complex and was shown to bind to Hsp90 in an ATP-dependent manner^6,17,18^. Subsequently, it has been shown that p23 is one of the few co-chaperones that are of general importance for processing diverse client proteins^1,2^. Hsp90 is a conserved molecular chaperone involved in the maturation of a diverse client spectrum including disease-related proteins^19–21^. Each protomer of the dimeric protein consists of three domains: the N terminal domain (NTD), which harbors the nucleotide-binding site, the middle domain (MD) responsible for completion of the split-ATPase as well as for client binding, and the C terminal domain (CTD), which is required for dimerization and TPR co-chaperone binding^22–25^. Similar to other chaperones^26,27^, Hsp90 is characterized by high conformational dynamics required for its activity: ATP binding to Hsp90 drives conformational rearrangements of the three domains to change the chaperone from an open/apo state^28^ via several intermediates to its closed conformation, in which the two NTDs are intertwined and the NTDs and MDs are twisted^13,29^. Importantly, this leads to the reorientation of a catalytic loop in the MD^24^. Notably, these conformational changes are modulated by a plethora of co-chaperones^30–33^. Once both NTDs are in close proximity, the p23 binding site is formed in which p23 is in contact with both Hsp90 monomers^13,34^. As p23 only binds to the closed conformation of Hsp90, specifically the closed-2 state^35,36^, it is considered as a conformational sensor of the Hsp90 cycle^17,18^. Interestingly, the stoichiometry of p23 binding is still unclear and both 1:1 and 1:2 (Hsp90_dimer_:p23) binding modes have been reported^37–41^. However, given that the concentration of p23 in the cell is much lower than that of Hsp90, binding of one p23 per Hsp90 forming an asymmetric complex seems physiologically relevant^35,42,43^. The binding of p23 to Hsp90 leads to a 50% reduction of the ATPase rate^39–41,44^ and to prolonged Hsp90 client association^41,45–48^.

Notably, early studies revealed that a minimal chaperone system of Hsp40, Hsp70, Hsp90, the adaptor protein Hop, and p23 are sufficient to chaperone clients in vitro^46,47,49,50^. Additionally, the relevance of p23 client maturation, in particular for steroid hormone receptors (SHRs) has been shown in mammalian cells and in yeast expressing SHRs^1,9,17,51,52^. In this context, p23 is one of the few co-chaperones of Hsp90 that is important for the maturation of all clients studied in a recent screen in yeast, which included different SHRs and v-Src kinase^1^. Apart from its role as an important Hsp90 co-chaperone, p23 has also been found to have Hsp90-independent functions such as the regulation of gene expression by modulating chromatin remodeling and ribosomal biogenesis^53,54^. Additionally, an Hsp90- and ATP-independent chaperone function has been reported^55,56^. For this, the unstructured p23 tail seems to be pivotal^12,15,16,57,58^.

Despite the importance of p23 and the progress made in the analysis of its structure and function over the years, we still lack an understanding of the mechanistic principles underlying the function of p23. The combination of a long unstructured segment together with a folded domain in a monomeric protein is unique among the co-chaperones. To gain insight into the interplay of these modules and their effects on Hsp90 function, specifically in the context of client maturation, we investigated structural features, dynamics, and molecular interactions by NMR and studied client activation in vivo using a variety of p23 mutants. Unexpectedly, we identify two specific elements in the tail that, together with the folded domain of p23, regulate the Hsp90 ATPase and mediate interaction with clients, respectively. These elements are both conserved between yeast and man.

## Results

### Dissection of the C-terminal tail of yeast p23

**Fig. 1:**
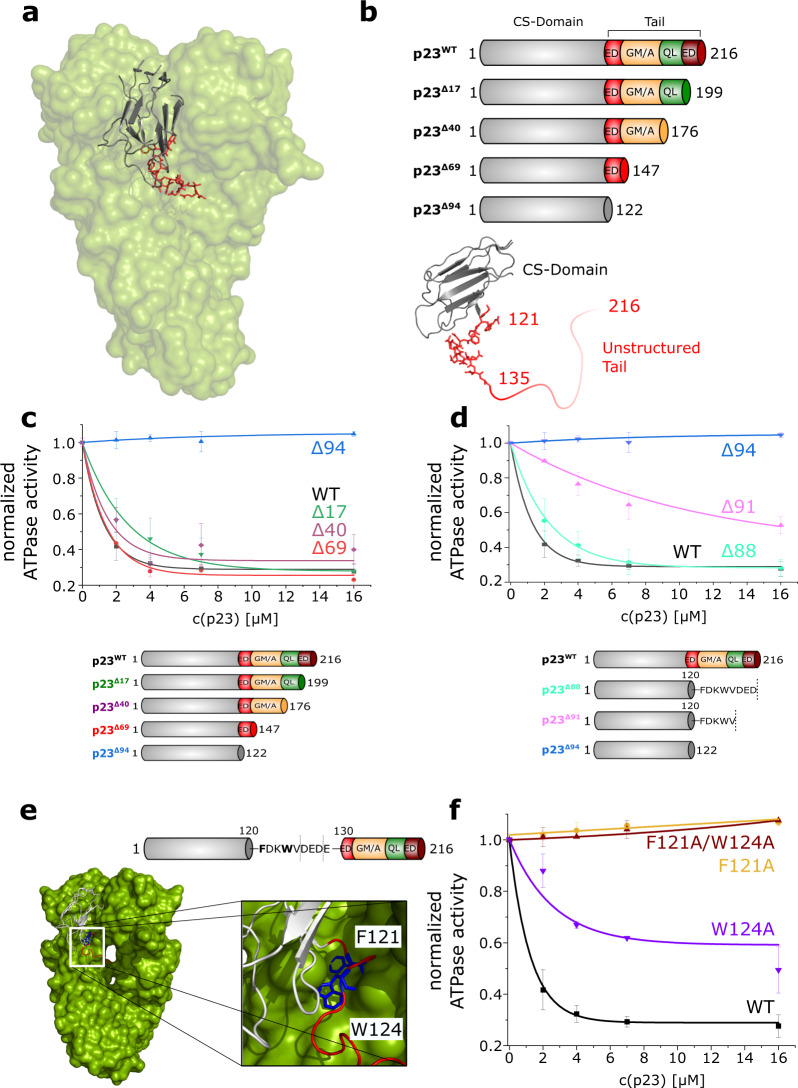
p23-mediated inhibition of the Hsp90 ATPase activity depends on F121 and W124. **a** The crystal structure of yeast p23 (Sba1) bound to yeast Hsp90 (Hsp82) is shown (PDB: 2CG9). The p23 CS domain is depicted in gray, the part of the p23 tail that was resolved in the crystal structure is shown in red. **b** The schematic domain architecture of p23 and the truncation mutants used in this study are shown. A close-up view of the p23 molecule is shown at the bottom (PDB: 2CG9). ED = negatively charged stretch; GM/A = GM/A-rich region; QL = QL-rich region; ED = second negatively charged stretch. **c**–**f** All subfigures show ATPase assays in the presence of WT or mutant yeast p23 (Sba1). The ATPase activity of yeast Hsp90 (Hsp82) was determined in the presence of rising p23 concentrations. WT (black) is shown as a reference in all figures. **c**, **d** Effects of the deletion mutants p23^Δ17^, p23^Δ40^, p23^Δ69^ and p23^Δ94^ on the ATPase activity of Hsp90. Note that the same data set was used for p23^WT^ and p23^Δ94^ in (**c**) and (**d**) as a reference. **e** Top: the p23 domain architecture is shown and the F^121^DKW^124^VD motif is highlighted. Bottom: crystal structure of p23 (gray cartoon) bound to the Hsp90 dimer (green surface) (PDB: 2CG9). The p23 CS domain is shown in gray, the tail region (up to aa 135) is depicted in red and the F121 and W124 residues are shown in blue. **f** Effect of the point mutants p23^F121A^, p23^W124A^ and the double mutant p23^F121A/W124A^ on the inhibition of the Hsp90 ATPase. Note that the WT data from (**c**) and (**d**) were plotted as a reference. Bars and errors represent means ± SD of triplicate measurements.

p23 is special among chaperone proteins as it contains a long disordered C-terminal tail that mediates its chaperone function^12,15,56^. To investigate the structure-function relationship of the 93 amino acid long tail, we first divided yeast p23/Sba1 according to the crystal structure^13^ in the folded core domain (CS domain) ranging from aa 1–122 and an unstructured tail from aa 123–216 (Fig. 1a,b). We subdivided the tail further into four motifs based on the amino acid composition (Fig. 1b). Adjacent to the core domain there is a small patch rich in negatively charged amino acids (aa 123–139, red), followed by a GM/A-rich region (aa 140–176, yellow) and a small QL-rich region (aa 177–199, green). At the C-terminal end of the tail there is a second highly negatively charged motif (aa 200–216, dark red). To test the influence of the different motifs on the functions of p23, we created truncation mutants in which one (p23^Δ17^) or more (p23^Δ40^, p23^Δ69^) of the motifs or the entire tail (p23^Δ94^) were deleted (Fig. 1b).

### A motif adjacent to the p23 core domain is important for the inhibition of the Hsp90 ATPase

As p23 is a known inhibitor of the Hsp90 ATPase^39,40,59^, we investigated how the different tail motifs influence the ATP turnover of yeast Hsp90 (Hsp82). Deletion of the C-terminal motifs in p23^Δ17^, p23^Δ40^ and p23^Δ69^ did not impair p23’s ability to partially inhibit the ATPase of Hsp90 (Fig. 1c). All mutants decelerated the Hsp90 ATPase with an apparent inhibitory constant K_i_ of 1.1–1.5 µM. By contrast, the folded p23 CS domain alone (p23^Δ94^) did not affect the ATP turnover at any concentration tested (Fig. 1c). This suggests that the region from aa 123–140 in p23 is essential for p23’s ability to inhibit the ATPase of Hsp90. To further query the region responsible, we generated two additional deletion mutants, p23^Δ91^ and p23^Δ88^. The p23^Δ88^ mutant nearly reached the inhibitory potential of the p23^WT^ with a K_i_ of 2.2 µM. p23^Δ91^, on the other hand, gave a K_i_ of 10.8 µM and thus has lost most of its inhibitory potential (Fig. 1d). From these results, we conclude that the region responsible for the ATPase inhibition lies in a region of the tail, which is resolved in the crystal structure of Hsp90 and p23^13^. Within the resolved stretch of the tail, F121 and W124 bind to a hydrophobic pocket of Hsp90 (Fig. 1e). To investigate the importance of these residues in more detail, the single point mutations (p23^F121A^ and p23^W124A^) as well as the double mutant p23^F121A/W124A^ were designed and tested for their effects on the Hsp90 ATPase. Introduction of the F121A mutation or F121A/W124A double mutation led to a complete loss of p23’s ability to inhibit Hsp90 (Fig. 1f). By contrast, the p23^W124A^ variant exhibited only a modest effect on the inhibitory constant (K_i_: 2.6 µM), but the inhibitory potential was compromised (Fig. 1f). Together, these results show that p23 F121 is essential for the inhibition of the Hsp90 ATPase and that W124 contributes to the inhibitory potential.

**Fig. 2:**
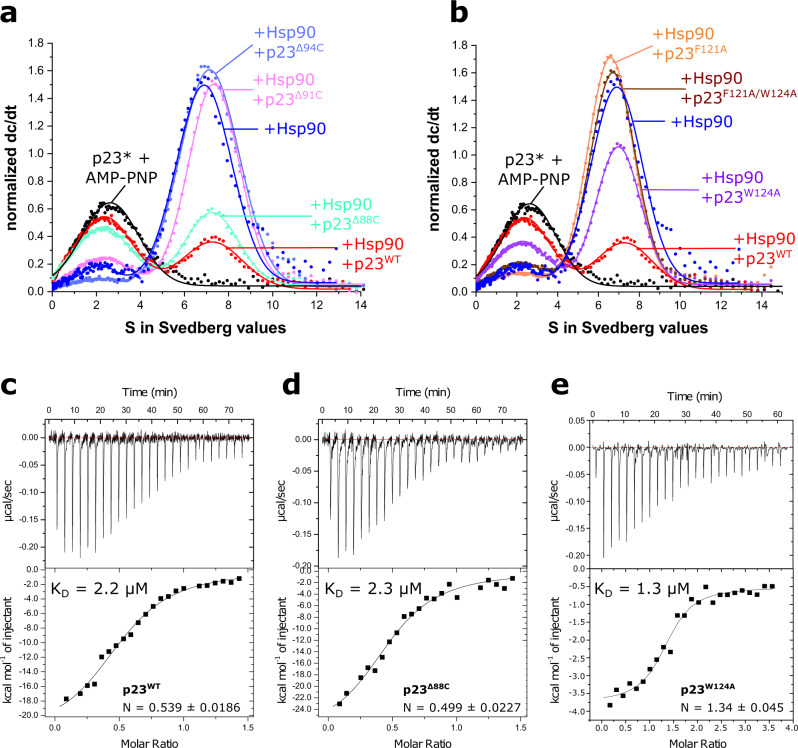
Analysis of the binding of p23 to Hsp90. **a**, **b** Analytical ultracentrifugation analysis of the binding between yeast Hsp90 (Hsp82) and yeast p23 (Sba1) mutants. All measurements were performed in the presence of 2 mM AMP–PNP. Labeled p23 (p23*) was bound to Hsp90 and an excess of the depicted p23 mutants was added. **c**–**e** Isothermal titration calorimetry analysis of the affinity between yeast (**c**) p23^WT^, (**d**) p23^Δ88^ and (**e**) p23^W124A^ and yeast Hsp90 (Hsp82). The measurements were performed in the presence of AMP–PNP. The shown curves are representatives of at least two replicates.

To test the extent to which the two residues contribute to the binding of p23 to Hsp90, we assayed deletion mutants as well as the point mutants for their ability to displace fluorescently labeled yeast p23^WT^ (marked with an asterisk: p23*) from a preformed yeast Hsp90 (Hsp82)-p23*-complex by analytical ultracentrifugation (aUC) (Fig. 2a, b). p23^WT^ and p23^Δ88^ were the only variants that replace the labeled p23 in a comparable manner, while p23^Δ91^ and p23^Δ94^ were largely inactive (Fig. 2a). Additionally, neither the p23^F121A^ nor p23^F121A/W124A^ mutants were able to efficiently compete p23* from Hsp90 (Fig. 2b). For p23^W124A^, a partial displacement was observed. These results imply that the missing ATP inhibition could be due to a defect in binding (Fig. 1f). Analysis of the affinity between Hsp90 and p23 mutants using isothermal titration calorimetry (ITC) gave comparable equilibrium dissociation constants (*K*_D_) of 2.2 μM and 2.3 μM, for p23^WT^ and p23^Δ88^, respectively (Fig. 2c,d). This agrees with the aUC analysis. The affinity of the p23^W124A^ mutant was similar (*K*_D_ = 1.3 µM), consistent with the ability to partially compete p23* from Hsp90 (Fig. 2e). By contrast, for p23^F121A^, the double mutant p23^F121A/W124A^ and the deletion mutant p23^Δ94^, no binding to Hsp90 could be detected by ITC (Supplementary Fig. 1). We conclude that the W124 residue plays a unique role in p23 function, with a similar binding affinity and inhibitory constant but reducing the maximum ATPase inhibition by 50%. Notably, both F121 and W124 are conserved between human and yeast p23.

### The p23 tail interacts with the core domain and contains helical segments

**Fig. 3:**
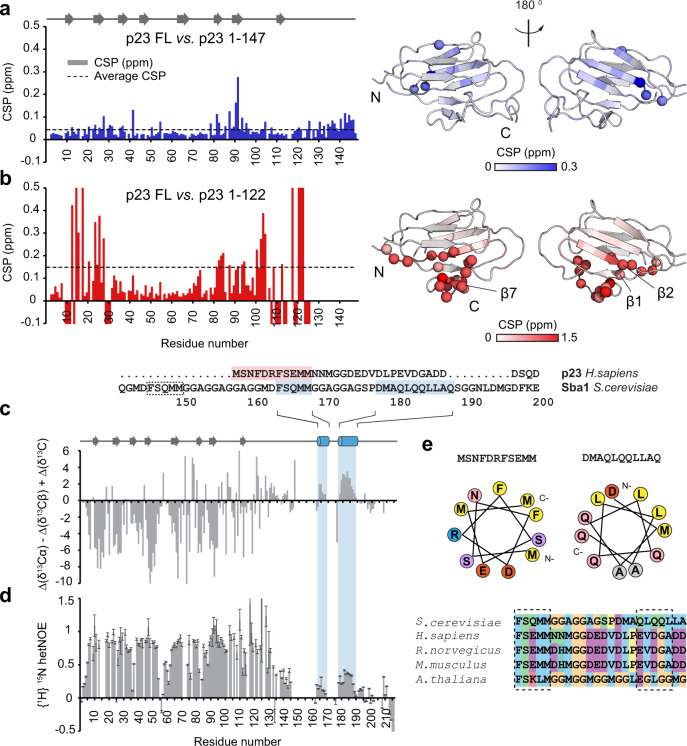
NMR analysis of different constructs of p23. Comparing chemical shift differences of the deletion mutants show that the p23^Δ69^ (1-147) mutant (**a**) is largely unaffected by the truncation of the tail. However, (**b**) the p23^Δ94^ mutant (1-122) reveals significant changes of the chemical shifts. Residues with a negative value indicate NMR signals which are absent in the NMR spectra of the tail-deleted construct. The spectral changes associated with tail deletions are plotted on the crystal structure of p23/Sba1 shown on the right. Color saturation indicates the strength of the chemical shift perturbation. Residues undergoing large chemical shift changes are shown as spheres. (**c**) Secondary ^13^C chemical shifts comparing differences in the C_α_, C_β,_ and C′ values versus random coil values of p23^WT^ (1–216). Positive and negative secondary chemical shifts are indicative of α-helical and β-strand conformation, respectively. Secondary structure elements are indicated at the top and highlighted in blue for yeast. In addition, the sequence alignment of human p23 is included, with the helical motifs highlighted in blue (yeast) and red (human). An additional predicted helical motif in yeast is indicated by a dotted box. **d** {^1^H}-^15^N heteronuclear NOE values obtained from the ratio of peak intensities of saturated vs. non-saturated experiments. Values around 0.78 correspond to rigid regions. Lower values correspond to increasing flexibility at sub-nanosecond timescales. Errors were estimated from spectral baseplane noise RMSD according to Farrow et al.^89^. **e** Polarity distribution of identified helical segments reveals their amphipathic properties. Orange/yellow colors indicate hydrophobic residues, while red/pink/blue colors represent charged amino acids. The sequence alignment shown at the bottom indicates the conservation of helical motifs in the p23 tail across different species, colored according to the Clustal code.

To better understand the structure and dynamics of the p23 tail, we employed NMR spectroscopy. We investigated potential interactions of the tail with the p23 core domain by comparing NMR spectra of three p23 constructs of varying tail lengths: p23^WT^, p23^Δ69^ (aa 1–147), and p23^Δ94^ (aa 1–122) (Fig. 1b). While the overall spectra of the constructs are relatively similar, many NMR signals are affected when parts of the tail are removed. p23^Δ69^ exhibits only minor chemical shift differences compared to the p23^WT^ spectrum (<0.3 ppm) (Fig. 3a), suggesting that this tail region does not interact significantly with the structured core domain. Removal of the remaining part of the tail in the p23^Δ94^ construct resulted in significantly more chemical shift changes (Fig. 3b). The most affected regions are the β1 strand (approximately aa 10–17), the C-end of β2 (aa 22–30) and residues 105–122 including β7 of the CS domain (Fig. 3a, b). This points to significant interactions of the tail fragment between aa 123–147 with the CS domain, suggesting that this part of the p23 tail forms contacts with the core domain.

Next, we focused on the secondary structure content of the p23 tail. We observed that amide signals of residues in the C-terminal tail exhibit little chemical shift dispersion, which indicates that the tail is disordered. Both the secondary ^13^C chemical shifts of Cα, Cβ, and C′ nuclei (Fig. 3c) and {^1^H}-^15^N heteronuclear NOE (hetNOE) measurements (Fig. 3d) confirm that the tail is highly flexible at sub-nanosecond timescales, consistent with a random coil conformation. Of note, high hetNOE values indicating a rigid conformation are observed for the N-terminal region of the tail up to residue 130 (Fig. 3d), which includes the 121-**F**DK**W**VDEDF-129 motif (Fig. 3c). Consistent with the large perturbations observed upon removal of tail residues 123–147, this shows that this region has limited flexibility likely due to interactions with the p23 core domain, although this region is not observed in the crystal structure of isolated human p23^12^.

Strikingly, we observed another region in the tail, from residues 178 to 188, that adopts a helical secondary structure (Fig. 3c, d). This section is significantly rigid based on hetNOE values and adopts an α-helical conformation based on ^13^C secondary chemical shifts, consistent with sequence-based predictions (Supplementary Fig. 2a). A second, shorter segment with helical propensity is observed between residues 164–168. Interestingly, an additional region predicted to form an amphipathic helix is present (Fig. 3c). To test whether the presence of the helical motif in the p23-tail is evolutionary conserved, we analyzed available NMR data of human p23^60^ and acquired additional data to assign ^13^C’ chemical shifts and characterize the dynamics based on hetNOE values. These data show the presence of a stretch with helical conformation comprising residues 117 to 127 in human p23 (Supplementary Fig. 2b), consistent with previous suggestions^16^. Thus, helical motifs are found in both human and yeast p23, suggesting an important function, confirming the evolutionary conservation of an amphipathic α-helical segment in the largely unstructured p23 C-terminal tail (Fig. 3e). In conclusion, while the majority of the p23 tail is unstructured, we found (i) that the conserved functionally important **F**DK**W**VD motif interacts with the folded domain of p23 and (ii) identified the presence of a conserved α-helical motif in the p23 tail.

### p23 affects specifically the NTD-MD arrangement of Hsp90 in the closed conformation

In the crystallographic structure of the complex between p23 and nucleotide-bound closed Hsp90^13^, the core domain binds to a groove at the junction of the two NTDs of Hsp90, together with the catalytic loop of the MD and several elements located at the NTD-MD interface. Only the first residues of the C-terminal p23 tail are resolved in the structure, pointing towards the Hsp90 cleft. However, the p23 complex likely is dynamic and allows for conformational flexibility between the Hsp90 NTD and MD^61^. To better understand the interaction and the inhibitory mechanism, we set out to determine the effects of p23 on the conformation of full-length Hsp90, including potential contacts of the p23 tail with the chaperone.

In a first step, we determined Hsp90-p23 interacting regions by recording ^1^H-^15^N correlation spectra of ^15^N-labeled p23 in the presence of unlabeled Hsp90 NTD or MD. We observed a specific subset of p23 signals exhibiting chemical shift perturbations in the presence of the Hsp90 MD (Supplementary Fig. 3b,d), consistent with previous studies of the human orthologs^60^. This includes residues in the core domain, in strands β1, β3, and β8 and part of the p23 tail (residues 124–140), which is in line with our results that residues 120–130 are essential for Hsp90 binding. In contrast, only minor perturbations were detected when p23 was titrated with the NTD both in the absence and in the presence of nucleotide (Supplementary Fig. 3a). When observing the interaction using ^15^N-labeled Hsp90 domains, we found that p23 binding causes strong perturbations for regions of the MD, in agreement with the crystal structure and previous NMR studies (Supplementary Fig. 3c, d)^13,60^.

**Fig. 4:**
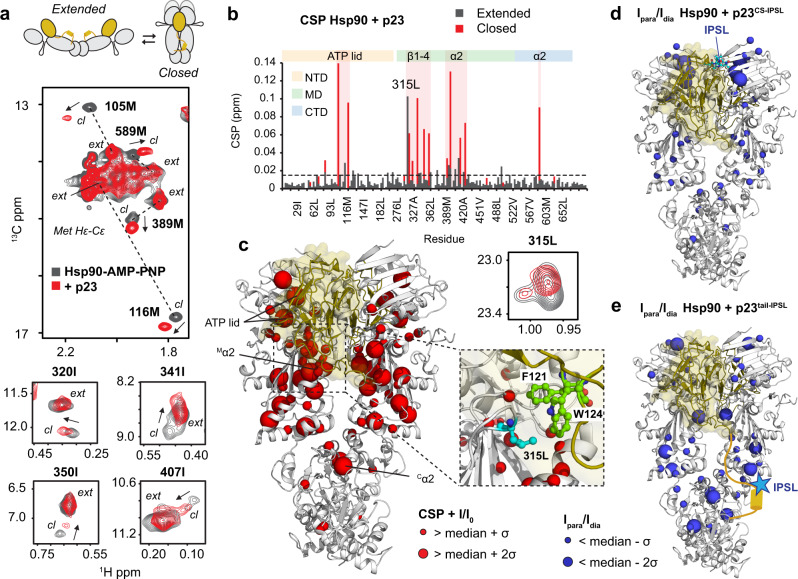
NMR Analysis of the interaction of p23 with Hsp90. **a** p23 triggers strong spectral changes for methyl signals in ^1^H-^13^C methyl-TROSY correlations. Top: schematic indicating open and closed conformations of the Hsp90 dimer with p23 CS and tail shown as yellow sphere and line. Middle: zoomed view of the methionine methyl region of the Hsp90-AMP–PNP spectrum in the absence (gray) and presence of 2.4 equivalents of p23 (red). Methyl signals corresponding to extended (*ext*) and closed (*cl*) conformations (in the presence of AMP–PNP) are connected by a dashed line. Chemical shift perturbations (CSP) induced by p23 binding that affect residues in the ATP lid, the helix α2 in the MD and the α2 helix projecting from the CTD are indicated by arrows. Bottom: CSPs induced by p23 binding for isoleucine methyl signals in the MD. Only the methyl signals corresponding to the closed conformation are affected by p23 binding. **b** CSP vs. residue number for methyl signals corresponding to extended and closed Hsp90 conformations upon binding of p23 (gray and red, respectively). Elements experiencing larger perturbations are highlighted in red and indicated at the top. **c** Perturbations and intensity changes are mapped onto the crystal structure of the Hsp90:p23 complex (PDB: 2CG9) as red spheres, p23 is shown in gold. A detailed view of the p23-Hsp90 interface is included on the right, indicating the most affected F121 and W124 residues of p23 (green) and L315 of Hsp90 (cyan). Zoomed view of L315 methyl resonance is shown at the top. **d** Intermolecular PRE of the complex with p23 spin labeled at residue 35 in the CS domain (Cys35-IPSL, cyan spheres). Residues experiencing line broadening are shown as blue spheres on the crystal structure. **e** PRE experiments using spin-labeled p23^C35A/S189C^ with IPSL conjugated to residue 189 flanking the helical motif in the p23 tail. Paramagnetic effects on several MD/CTD residues (blue spheres) indicate transient interactions with the tail helix. Experiments were performed at 100 μM of Hsp90 and p23 spin labeled at 2:1 ratio corresponding to 80% of a 2:1 complex of Hsp90:p23, according to the stoichiometry obtained by ITC. Only one paramagnetic center was used for the calculations by artificially removing one p23 chain. In order to account for the different paramagnetic effects on the two protomers, the intensity ratios for protomers A and B were averaged.

The use of methyl-labeled Hsp90 allowed us to analyze the binding of p23 to the full-length protein. Upon addition of p23 to the Hsp90:AMP–PNP complex, we observed chemical shift changes in the NTD and MD that are consistent with the crystal structure (Fig. 4a–c, Supplementary Fig. 4b)^13^. This is especially the case for L315, which is sandwiched between the F121-W124 aromatic pair of p23 and has previously been shown to lie in the p23 interacting surface^13^. Intriguingly, additional changes occur in the ATP-lid and for residues of the N-M interface. To investigate the structure of the complex in solution, we performed intermolecular paramagnetic relaxation enhancement (PRE) experiments, in which p23 was labeled with a paramagnetic tag at C35 of the CS domain^62,63^. As seen in Fig. 4d and Supplementary Fig. 5a, strong signal broadening occurs for residues in the N-terminus, α4 and β4 of the NTD, due to spatial proximity to the spin label, consistent with the crystallographic structure^13^. However, relaxation enhancements are significantly weaker compared to the theoretical values derived from the structure (Supplementary Fig. 5a). This indicates the presence of heterogeneous populations of the complex, with some states involving weaker contacts between p23 and the NTD of Hsp90.

Our NMR data show that binding of AMP–PNP induces the appearance of a second set of signals in the ^1^H-^13^C methyl-TROSY spectrum (Fig. 4a, Supplementary Fig. 4a). Since the binding of AMP–PNP induces the closing of Hsp90^29,64^, these signals indicate the presence of a minor population that likely represents a closed conformation in slow equilibrium with an ensemble of extended states^65,66^. Interestingly, p23 induced strong chemical shift changes and PRE effects exclusively for the second set of signals, indicating that p23 binds more tightly and largely affects the closed Hsp90 conformation (Supplementary Fig. 4a, 5). The residues affected cluster in the ATP-lid of the NTD, the catalytic loop, the N-terminus of helix α2 and the adjacent strands β1-4 of the MD, as well as the amphipathic helix α2 of the CTD projecting into the dimer cleft (Fig. 4c). To further validate these results, we performed p23 titration in the presence of the physiological ATP. The high similarity of chemical shifts corresponding to the closed state corroborates the specific effects of p23 on this conformation (Supplementary Fig. 6a). NMR titrations using the p23^W124A^ mutant, which has reduced Hsp90 inhibitory capacity, show similar shifts except for the residues at the N-terminal end of helix α2 of the Hsp90 MD. Notably, the chemical shifts of these signals are close to those of the unbound Hsp90 MD (Supplementary Fig. 6b). Taken together, our results indicate that nucleotide-bound Hsp90 adopts a dynamic ensemble of closed and open states. p23 specifically binds to the closed conformation and thereby induces changes in the NTD-MD arrangement of the closed states involved in catalysis. As a result, the active configuration of the catalytic loop and α2 of the MD is shifted from the hydrolytic state and interfering with the activity (Supplementary Fig. 6c)^13^.

Interestingly, NMR titrations of p23 to ^13^C methyl-labeled, deuterated full-length Hsp90 dimer revealed chemical shift perturbations in the MD and CTD that are remote from the interaction site with the p23 core domain. We hypothesized that this reflects transient interactions of the p23 tail. To test if the helical element that we identified in the p23 tail is responsible for these interactions, and confirm that this involves direct contacts, we analyzed PREs with a spin-labeled mutant C35A/S189C of p23 containing a Cys residue right after the helical stretch of the C-terminal tail. As seen in Fig. 4e and Supplementary Fig. 5b, PRE effects concentrate on the MD, especially at exposed sites of helices α2, α3, and α6. On the CTD, tail contacts occur with the amphipathic helix α2, which projects into the internal side of the Hsp90 dimer, an important recognition site for steroid hormone receptors^65,67,68^. Additional weak PRE effects observed on the NTD reflect transient tail contacts due to the dynamic interaction of the p23 tail with the MD-CTD region.

### Interaction of p23 with the GR

**Fig. 5:**
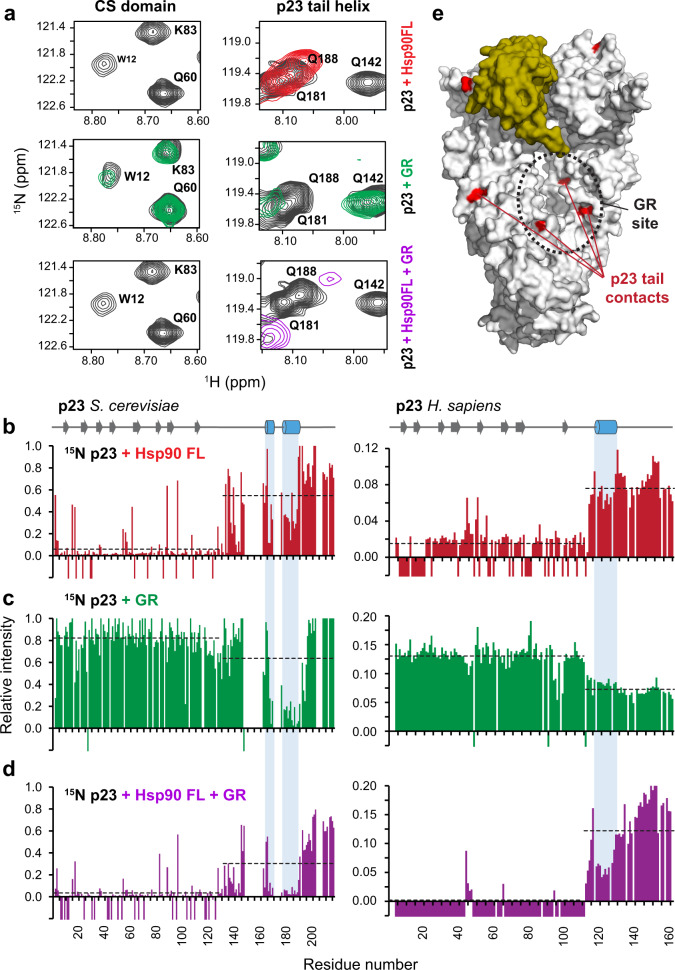
NMR analysis of ^15^N-labeled p23 in the presence of Hsp90/AMP–PNP, and/or GR. **a** Zoomed view of ^1^H-^15^N HSQC NMR signals in the CS domain and the helical motif in the p23 tail comparing p23 free in solution (black), in the presence of Hsp90 FL (red), GR-LBDm (green) or Hsp90 FL and GR-LBDm (purple). Intensity ratios of p23 peaks in the absence and in the presence of (**b**) full-length Hsp90 (FL), (**c**) the GR ligand binding domain (GR-LBDm), or (**d**) Hsp90 FL and GR-LBDm, for yeast (left) and human (right). Negative bars indicate resonances broadened beyond detection; the average peak intensity for the p23 core domain and the C-terminal tail is shown as a dashed line. Secondary structure elements are shown at the top, highlighting the helical motifs in the p23 tail. **e** Binding sites of the p23 tail and GR interactions with the Hsp90 dimer overlap at the MC interface.

It has been shown that p23 is an important component required for the maturation of the Hsp90 client GR^1,49,52,69,70^. To better understand the role of p23 in this process, we sought to investigate whether there is an interaction between p23 and GR. In a first step, we titrated ^15^N-labeled p23 with the Hsp90 dimer bound to AMP–PNP. As expected, signals in the p23 core domain experience severe line broadening upon complex formation, and additional line broadening is seen for the helical motif in the p23 tail, consistent with transient contacts with Hsp90 MC (Fig. 5a,b). When we added the GR ligand binding domain (LBD) to labeled p23 in the absence of Hsp90, we also observe a significant reduction of peak intensities in the tail corresponding to the α-helical region between residues 178–188, and to a minor extent, to the preceding helix 164–168 (Fig. 5a, c). This demonstrates that the helical motif directly contacts the GR client. When we formed the ternary p23-Hsp90–GR complex, we observed an additional reduction of the intensities of peaks corresponding to regions in both the core domain and the helical tail (Fig. 5a, d). This indicates that in the p23-Hsp90–GR complex the p23 tail directly contacts the GR client, as the GR occupies the region in Hsp90 MC, which transiently interacts with the p23 tail in the absence of the client. Interestingly, similar results are obtained for human p23. Similarly, the helical segment that we identified between residues 117–127 in human p23 shows spectral changes upon binding to GR-LBD or Hsp90β, and in the ternary complex (Fig. 5b–d, right). To demonstrate that the helical structure in the tail of p23 is necessary for binding, we introduced the helix-breaking L182P substitution and analyzed the secondary structure and interactions of this mutant. Secondary ^13^C chemical shifts and hetNOE values show that this substitution effectively disrupts the helix (Supplementary Fig. 7a, b). Importantly, complex formation with the ^15^N-labeled p23 L182P mutant and Hsp90 or GR shows no significant spectral changes in the p23 tail, stressing the crucial role of an amphipathic helix for productive binding (Supplementary Fig. 7c–e). Together, this indicates that p23 harbors a helical motif in the tail that transiently interacts with Hsp90 and mediates direct contacts to the GR client, in both yeast and humans. Importantly, the p23 tail helix interacts with the internal cleft of Hsp90 and the helix 2 in the CTD, which are both important sites for steroid hormone receptor binding (Fig. 5e). Thus, p23 may scan the Hsp90 surface for the presence of a client and guide the client to the binding site on the Hsp90 dimer.

### The p23 tail is important for GR maturation in vivo

**Fig. 6:**
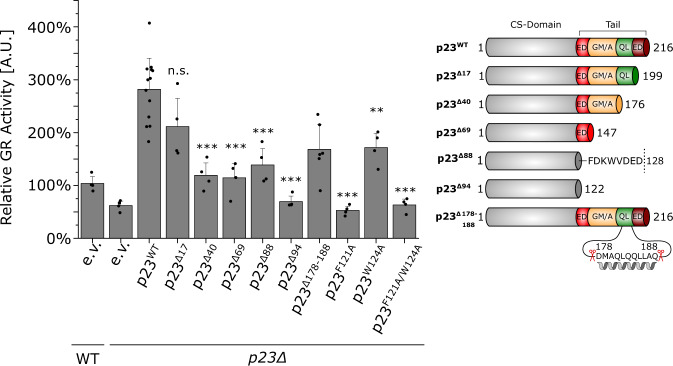
The p23 tail is required for GR folding in vivo. Wild-type or *sba1Δ* yeasts cells were examined concerning their ability to mature heterologously expressed glucocorticoid receptor (GR). Cells either harbored the empty vector (e.v.) plasmid as control or expressed the indicated p23 mutants. The relative GR activities compared to the wild-type control are shown. Bars represent means ± SD of at least four independent experiments. Significance was evaluated by two-tailed t-testing (n.s. *P* ≥ 0.05; *P ≤ 0.05; **P ≤ 0.01; ***P ≤ 0.001) and Welch correction was used if variances were significantly different between samples. [p(p23^Δ17^) = 0.06, p(p23^Δ40^) = 0.00014, p(p23^Δ69^) = 0.00012, p(p23^Δ88^) = 0.00054, p(p23^Δ94^) = 9.6 × 10^−9^, p(p23^Δ178-188^) = 0.00126, p(p23^F121A^) = 5.0 × 10^−9^, p(p23^W124A^) = 0.004, p(p23^F121A/W124A^) = 6.2 × 10^−9^].

The observed interactions of the p23 tail with Hsp90 and the GR-LBD prompted us to investigate its influence on GR activation in vivo. To this end, we tested the effect of p23 tail truncations on GR activation in *S. cerevisiae* (Fig. 6). The analysis of GR in yeast as a client for the Hsp90 system via reporter assays is well established^52,67,70–72^ and GR activation specifically requires p23 function as a knockout of p23/Sba1 reduced GR activity to about half of WT activity^1,52^. We overexpressed p23/Sba1 in the *sba1Δ* strain from a plasmid under the strong constitutive GPD promoter. Accordingly, we observed a 2.5-fold activation of GR activity compared to the endogenous p23 levels suggesting that p23/Sba1 promotes GR activation in a concentration-dependent manner. In line with our ATPase experiments and binding studies, insertion of the F121A mutation and the F121A/W124A double mutation abrogated GR activity entirely. The W124A mutant, which binds to Hsp90 and inhibits the ATPase to a smaller degree, displayed a less pronounced impairment of GR activation. Thus, the modulation of Hsp90 mediated by the interaction of F121 and W124 is crucial for GR activation in vivo.

Stepwise trimming of the C-terminal tail (p23^Δ17^, p23^Δ40^, p23^Δ69^, and p23^Δ88^) led to a gradual decrease in GR activity and the variant in which the entire tail (p23^Δ94^) was deleted behaved like the *sba1Δ* strain. Of note, in vivo the p23^Δ40^ (aa 1–176) and p23^Δ69^ (aa 1–147) deletion mutants displayed a pronounced reduction of GR activity compared to p23^WT^, while they behaved like p23^WT^ regarding Hsp90 binding and ATPase inhibition (Figs. 1,  2). By contrast, the expression of the p23^Δ17^ mutant did not significantly affect GR activity compared to p23^WT^ in vivo (Fig. 6). This is in agreement with our finding of the helical segment between the residues 178–188 as the GR-binding motif. Of note, deletion of just the predicted helical segment (p23^Δ178-188^) in the tail also significantly reduced GR activation in vivo. Thus, these results establish the importance of the α-helical region in the p23 tail for GR activation in vivo.

## Discussion

Recent studies revealed a central role of the chaperone p23 in the activation of Hsp90 clients in vivo^1^. In addition, it is well established that p23 plays important functions in chromatin organization and the DNA interaction of transcription factor complexes^53,54^. Structurally, p23 has a unique topology containing a folded core CS domain and a long C-terminal tail region. While it seems established that the tail plays an important role in the chaperone function of p23, the mechanistic basis has remained largely elusive. This is partly due to the lack of structural information for the tail^12,13^. In this study, we unraveled the “active sites” in p23 and determined structural features of p23 as well as their contribution to its biological function and to the mechanism of Hsp90 ATPase inhibition.

Our results show that the residues F121 and W124 at the beginning of the tail play a pivotal role for the binding of yeast p23 to Hsp90 in line with findings for human p23, in which the homologous F103A and W106A mutations abolished Hsp90 binding^73^. We were able to differentiate between the function of the two aromatic residues and found that the W124A mutant still binds to Hsp90 but the ATPase inhibition is diminished. Thus, specifically W124 is responsible for inhibiting the ATPase of Hsp90. In the crystal structure of Hsp90 in complex with p23, F121 and W124 bind to a hydrophobic patch of the Hsp90 MD^13^. Notably, the W124 side chain interacts with several residues of the N-terminal region of helix α2 and the catalytic loop of the MD, which show two conformations when bound to the nucleotide. The presence of different conformations in these elements was observed in the crystal structures of the isolated MD and the FL protein^13,24,74^. Consistent with our activity assays and NMR data for p23 mutants, we propose that W124 interactions are critical to shift the active conformation of catalytic elements of the MD, while F121 provides the driving force for binding. The negatively charged residues adjacent to W124 might contribute to stabilizing electrostatic interactions with Lys residues 387 and 390 of the helix α2 of the domain. Hence, we conclude that, at least part of the inhibitory mechanism of p23 is to induce changes in structural elements of the NTD-MD interface of a closed Hsp90 dimer, which are involved in ATP hydrolysis. Yeast p23 has been found to bind exclusively to the ATP-bound, hydrolysis-competent state of Hsp90^40^. The inhibitory mechanism of p23 is still unclear and two basic concepts are discussed: either binding of p23 prevents ATP hydrolysis in Hsp90, or hydrolysis takes place and the release of ADP + Pi is inhibited^75^. Our data support a model in which p23 inhibits ATP hydrolysis by shifting the catalytic loop and helix α2 of the MD. As this shift occurs with both ATP and non-hydrolysable analogues, p23 inhibition may occur in a pre-hydrolysis step.

**Fig. 7:**
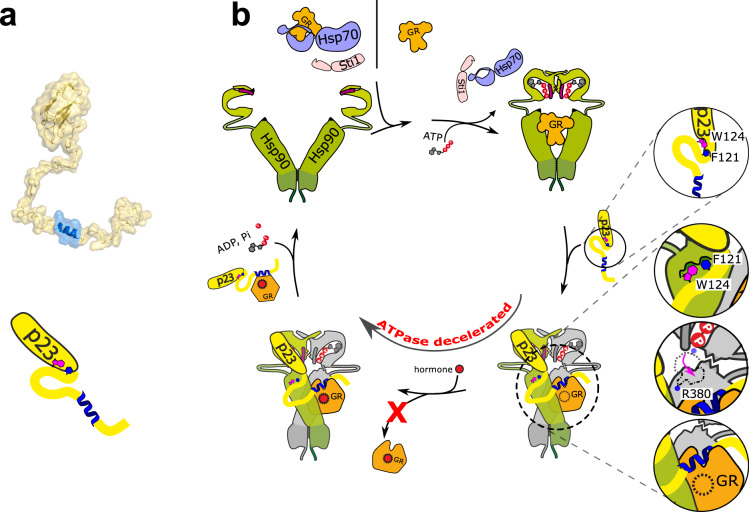
Schematic model of p23 function in the Hsp90 chaperone cycle. **a**. Model of p23. The p23 tail has been modeled to the CS domain of yeast p23 (PDB: 2CG9) using PyMol 1.7.1.1. The tail helix is shown in blue. A schematic model is shown at the bottom. **b**. The GR either binds Hsp90 directly or is recruited from Hsp70 to Hsp90 via the adapter co-chaperone Sti1/Hop. PPIases compete with Sti1/Hop for Hsp90 binding and displace Sti1/Hop (not shown). In free p23, the W124 and F121 residues contact the core domain (1st inset). Upon binding to the ATP-bound, closed Hsp90 conformation, these aromatic residues bind a hydrophobic pocket on the Hsp90 middle domain (2nd inset). The inhibitory effect of p23 on the Hsp90 ATPase is caused by a shift of the catalytic loop conformation, which positions the R380 residue in a way that prevents ATP hydrolysis (3^rd^ inset). The C-terminal tail of p23 contains a helical segment, which interacts and stabilizes Hsp90-bound GR and prevents premature dissociation of the GR from Hsp90 (4^th^ inset).

The binding of p23 to a specific closed conformation of Hsp90 allows positioning this co-chaperone at a specific point of the chaperone cycle, at which the client is engaged with Hsp90. Previous studies suggested that p23 serves to stabilize client complexes^46,47,49^. This could rely solely on the inhibition of the ATPase thus stabilizing the closed, client-engaging state. If this was the case, then truncated versions of p23 lacking the tail but retaining W124 should be fully active in client processing. However, we observe that GR maturation is decreased in the presence of truncated versions of p23 in yeast. Specifically, the helical region in the unstructured tail is important in this context (Fig. 7). This suggests a direct interaction of this helix with Hsp90 and/or with the client. We provide experimental evidence that this helix forms transient contacts with an amphipathic helix protrusion in the Hsp90 MD/CTD, which has previously been shown to be important for client binding (Fig. 7)^20,65,67,68,76,77^. Importantly, this helix is conserved between yeast and human p23. Additionally, our NMR data show that the helix is involved in direct interactions with the GR-LBD, which may stabilize the GR even in the absence of Hsp90, consistent with Hsp90-independent chaperone functions of p23 (Fig. 7)^55,56^. Given that the transient interactions of the p23 tail with the Hsp90 dimer in the absence of the client map to the client binding site, the p23 tail may guide and regulate client binding to Hsp90. The interactions of the p23 tail with the client while bound to Hsp90 may contribute to stabilizing a conformation of GR-LBD and establish the topology of the ternary complex (Fig. 7) in line with structural features of the ternary Hsp90:GR-LBD:p23 complex^78^. Since the client spectrum of Hsp90 is diverse spanning structurally unrelated kinases, E3 ligases, protein kinases, and transcription factors among others^79^, the amphipathic helix in the p23 tail can be envisioned to engage with different clients. This is also in line with the finding that p23 has been found as a general regulator of various clients in vivo^1^. In this scenario, the long unstructured tail would allow fulfilling the requirement of combining the precise positioning of the folded regulatory p23 CS domain at the NTD with the flexibility required to engage with nonnative segments of diverse clients positioned at different distances. In this context, considering that according to our NMR data additional regions in the largely disordered tail exhibit reduced flexibility and helical propensity, these motifs could be engaged in the interaction with different clients consistent with the general importance of p23 for client processing by Hsp90 and its Hsp90-indendent functions.

## Methods

### Protein expression, purification, and labeling

p23 mutants were cloned using the primers shown in Supplementary Table 1 using Q5 site-directed mutagenesis (New England Biolabs, Massachusetts, USA). Hsp90 and p23 (wild type and variants) were expressed and purified as previously described^35,80^. Briefly, Hsp90 and p23 mutants were expressed in E. coli BL-21 DE3 by induction with 1 mM isopropyl-β-D-thiogalactopyranoside (IPTG) at an OD_600_ of 0.8 and expression at 37 °C for 4 h while shaking. Cells were lysed by pressure and the lysate was cleared by centrifugation at 45,000*g* for 1 h. Hsp90 and mutants were then purified using affinity-chromatography, ion exchange chromatography, and size exclusion chromatography. To produce isotope-enriched proteins, the LB media was replaced with M9 minimal media supplemented with 2 g of ^13^C D-glucose and/or 1 g ^15^N ammonium chloride as the sole source of carbon and nitrogen. Cells used for deuterated protein were grown in 99.8% D_2_O (Sigma-Aldrich) M9 medium, after adapting pre-cultures in 0, 50%, and 99.8% D_2_O steps. The final culture was supplemented with 2 g ^2^H,^13^C D-glucose as the sole source of carbon. Precursors for stereospecific Ala, Ile, Met, Leu/Val *pro-*R labeling were added prior to induction with 1 mM IPTG according to the times specified for the supplier (NMR-bio, Grenoble, France). Expression takes place overnight at 30 °C.

### Assignment of methyl-labeled full-length Hsp90

For the assignment, ILVM labeled individual domains were expressed in an otherwise deuterated background^81^ and methyl resonances were assigned by conventional backbone to side-chain experiments and 3D HCC NOESY experiments. Ala resonances were partially assigned from the C chemical shifts from HNCACB/HNcoCACB experiments and a combination of ^15^N and ^13^C edited NOESY experiments recorded with ^13^C, ^15^N-labeled samples. To minimize the overlap in Leu and Val signals in the full-length spectrum, stereospecific assignments of pro-*R* methyl groups was used^82,83^. To assign the ^1^H-^13^C methyl-TROSY spectrum of the full-length protein, assignments were transferred from the individual domains and complemented by 3D HCC NOESY experiments on the intact protein. Assignments were supported by comparison with the N-M construct and completed by mutagenesis of several residues lacking NOE networks. To assign the additional resonances of the closed state in the presence of AMP–PNP, the minimal chemical shift deviation approach was used for signals shifted less than ~0.1 ppm. In the case of the ATP lid and helix α2 of the CTD, chemical shift assignments were confirmed by mutagenesis.

### ATPase activity assay

ATPase activity assays were performed at 30 °C using an ATP-regenerating system as described elsewhere^84^. Assays were performed in assay buffer (40 mM Hepes, 150 mM KCl, 5 mM MgCl_2_, 1 mM DTT, pH 7.5). Briefly, 3 µM yeast Hsp90 (Hsp82) were mixed with different concentrations of yeast p23 (Sba1) and mutants of yeast p23 (Sba1) in a volume of 75 µL. 2x reaction premix (5.17 mM phosphoenolpyruvate, 0.43 mM NADH, 6 units of pyruvate kinase and 30.25 units of lactate dehydrogenase in assay buffer) was prepared and 75 µL of the premix were added to the p23-Hsp90 mix. The reaction was started by the addition of ATP to a final concentration of 2 mM and the absorption at 340 nm was measured over at least 20 min in a Varian Cary 50 (Varian, Palo Alto, USA) using the Cary WinUV software. Background activity was measured after the addition of 100 µM radicicol and subtracted. Shown are means ± SD of 3 biological replicates.

### Analytical ultracentrifugation (aUC)

To determine the binding of the respective yeast p23 (Sba1) mutants to yeast Hsp90 (Hsp82), sedimentation velocity aUC in a Beckman Coulter XL-A analytical ultracentrifuge (Beckman, Krefeld, Germany) equipped with a fluorescence detection system (Aviv, Lakewood, USA) was conducted. 0.5 µM of Atto488-labeled yeast p23 (Sba1) were incubated with 3 µM of yeast Hsp90 (Hsp82). Additionally, 6 µM of the unlabeled Sba1 mutants were added to compete for Hsp82 binding. For the interaction studies with labeled Sba1, 40 mM Hepes (pH 7.5), 150 mM KCl, 5 mM MgCl_2_, supplemented with 2 mM AMP–PNP (Cat. No. 10102547001, Roche, Basel, Switzerland) were used. Measurements were performed at 20 °C and 42.000 rpm in an An-50 Ti (Beckman, Krefeld, Germany) rotor. SEDVIEW was used for the data conversion into a dc/dt representation and data was further analyzed via Origin 2019, fitted with Gaussian equations.

### ITC measurements

The binding of yeast Hsp90 (Hsp82) and yeast p23 (Sba1) was measured in a MicroCal ITC 200 at 30 °C. The sample cell was filled with 20 µM Hsp90 in addition to 2 mM AMP–PNP (Cat. No. 10102547001, Roche, Basel, Switzerland) and the syringe with p23^WT^ (135 µM) or mutants (up to 360 µM) in addition to 2 mM AMP–PNP. Measurements took place in 40 mM Hepes, 150 mM KCl, 5 mM MgCl_2_, 1 mM DTT, pH 7.5 buffer. 25 injections were done and the released heat and binding affinities directly determined. Measurements were conducted in duplicates or triplicates.

### NMR Experiments

NMR spectra were recorded on Bruker AV800, 900, and 950 spectrometers (Bruker Topspin 3.5, Bruker, Karlsruhe, Germany) at 30 °C, processed using NMRPipe and analyzed using CcpNmr 2.4.2. Unless mentioned otherwise, NMR buffer used was 20 mM sodium phosphate, 100 mM NaCl, 5 mM EDTA, 0.2 % NaN_3_, pH 6.5 (NMR buffer). Due to the unstable nature of GR, experiments which involved GR binding used the buffer conditions optimal for GR stability, 25 mM Tris, 200 mM NaCl, 5 mM DTT, 0.5 mM Dexamethasone, 1% DMSO (deuterated), 0.2 % NaN_3_ pH 7. Labeled protein concentrations were in the range from 100 to 250 µM unlabeled binding components were always added to a minimum of 1.2 equivalents. AMP–PNP (Cat. No. 10102547001, Roche, Basel, Switzerland) and MgCl_2_ were added to a final concentration of 5 mM.

Chemical shift assignments are already available for both Hsp90-N domain (aa 1-210)^85,86^ and, M-domain (aa 277-527)^87^. Assignments for the segmentally ligated Hsp90-NM constructs were obtained previously^65^. Assignments for p23 were obtained through standard triple resonance assignment experiments.

Chemical shift perturbations (CSP) were based on 2D ^1^H,^15^N water-flip-back HSQC correlation experiments comparing the isotopically labeled component of interest in the presence or absence of the binding protein component at natural abundance and calculated as

1 $${\Delta}\delta _{N - H} = \sqrt {\left( {{\Delta}\delta _{^1H}} \right)^2 + \left( {\alpha \ast {\Delta}\delta _{^{15}N}} \right)^2}$$

where α is a scaling factor between ^1^H and ^15^N dimensions with a value of 0.16.

### In vivo GR activity

Activity of the GR in yeast was measured as published previously^1^. Briefly, wild-type (BY4741, MATa, *his3Δ1, leu2Δ0*, *met15Δ0, ura3Δ0*) or *SBA1*-Knockout (BY4741, MATa, *ura3Δ0*, *leu2Δ0*, *his3Δ1*, *met15Δ0*, *YKL117w::kanMX4*) *S. cerevisiae* cells were transformed with the p413GPD-GR and the reporter plasmid pUCΔSS-26X that drives LacZ expression in a GR dependent manner^88^. Different Sba1 mutants were constitutively expressed from the p415GPD plasmid. Cells were grown to stationary phase at 30 °C while shaking and the diluted 1:10 in fresh selective media supplemented with 10 µM 11-deoxycorticosterone (Sigma-Aldrich) grown overnight at 30 °C while shaking. Subsequently, 50–100 µl of cells were pelleted in a 96-well plate and permeabilized by incubation with 150 µl lysis buffer (Na_2_HPO_4_ 82 mM, NaH_2_PO_4_ 12 mM, 0.1% SDS, pH 7.5) while shaking at room temperature. The reaction was started by addition of ortho-Nitrophenyl-β-D-galactoside to a final concertation of 1 mg/ml. The kinetics of β-galactosidase activity was monitored at 420 nm and the linear region was used to calculate β-galactosidase activity. The activity was normalized to the OD_600_ signal of each well. Statistical significance was evaluated by two-tailed t-test using Origin 2019 (t-test n.s. *P* ≥ 0.05; *P ≤ 0.05; **P ≤ 0.01; ***P ≤ 0.001) and Welch correction was used in case variances were significantly different.

### Reporting summary

Further information on research design is available in the Nature Research Reporting Summary linked to this article.

## Supplementary information

Supplementary Information

Reporting Summary

## Data Availability

The NMR assignments of methyl labeled yeast Hsp90 have been deposited to the Biological Magnetic Resonance Bank (BMRB) under accession code 50474. Source data are provided with this paper. Other data are available from the corresponding authors upon reasonable request. Source data are provided with this paper.

## Source data

Source Data
