# Supplementary material for: Structural elements in the flexible tail of the co-chaperone p23 coordinate client binding and progression of the Hsp90 chaperone cycle
Source: Nat Commun. 2021 Feb 5;12:828. doi: 10.1038/s41467-021-21063-0 (PMC7864943; doi:10.1038/s41467-021-21063-0)
Supplement: Supplementary file 1 — Supplementary Information [file 41467_2021_21063_MOESM1_ESM.pdf]

Supplementary Table 1
Primer sequences used to clone p23 mutants

Primer Name	Sequence 5' - 3'
sba1-d17C_f	TAAATGTCCGATAAAGTTATTAAC
sba1-d17C_r	TTTGAAATCTCCCATGTC
sba1-d40C_f	TAAATGTCCGATAAAGTTATTAACCCTCAAGTTG
sba1-d40C_r	AGAACCAGCGCCACCAGC
sba1-d69C_f	TAAATGTCCGATAAAGTTATTAAC
sba1-d69C_r	GCTGAAATCCATTCCTTG
sba1-d88C_f	TAAATGTCCGATAAAGTTATTAAC
sba1-d88c_r	ATCTTCATCAACCCATTTATC
sba1-d91C_f	TAAATGTCCGATAAAGTTATTAAC
sba1-d91c_r	AACCCATTTATCGAAATCAG
sba1-d94C_f	TAAATGTCCGATAAAGTTATTAAC
sba1-d94C_r	ATCGAAATCAGTTTTGATGTAAG
sba1_F121A_F	CAAAACTGATgcgGATAAATGGGTTGATG
sba1_F121A_R	ATGTAAGGGTACTTCACC
sba1_W124A_F	TTTCGATAAAgcgGTTGATGAAGATGAAC
sba1_W124A_R	TCAGTTTTGATGTAAGGG
sba1-L182P_f	TATGGCTCAAcctCAGCAATTATTGGCTCAAAGCGG
sba1-L182P_r	TCTGGAGAACCAGCGCCA
sba1_S35A_f	AATTGCAGACgctGATGCCCCTGAG
sba1_S35A_r	GACACAGTTATTAAGACATAATTTC
sba1_S189C_F	ATTGGCTCAATGCGGTGGTAATT
sba1_S189C_R	AATTGCTGCAATTGAGCC
sba1_178_188_del_F	AGCGGTGGTAATTTGGACATGG
sba1_178_188_del_R	TGGAGAACCAGCGCCACC

Supplementary Figure 1

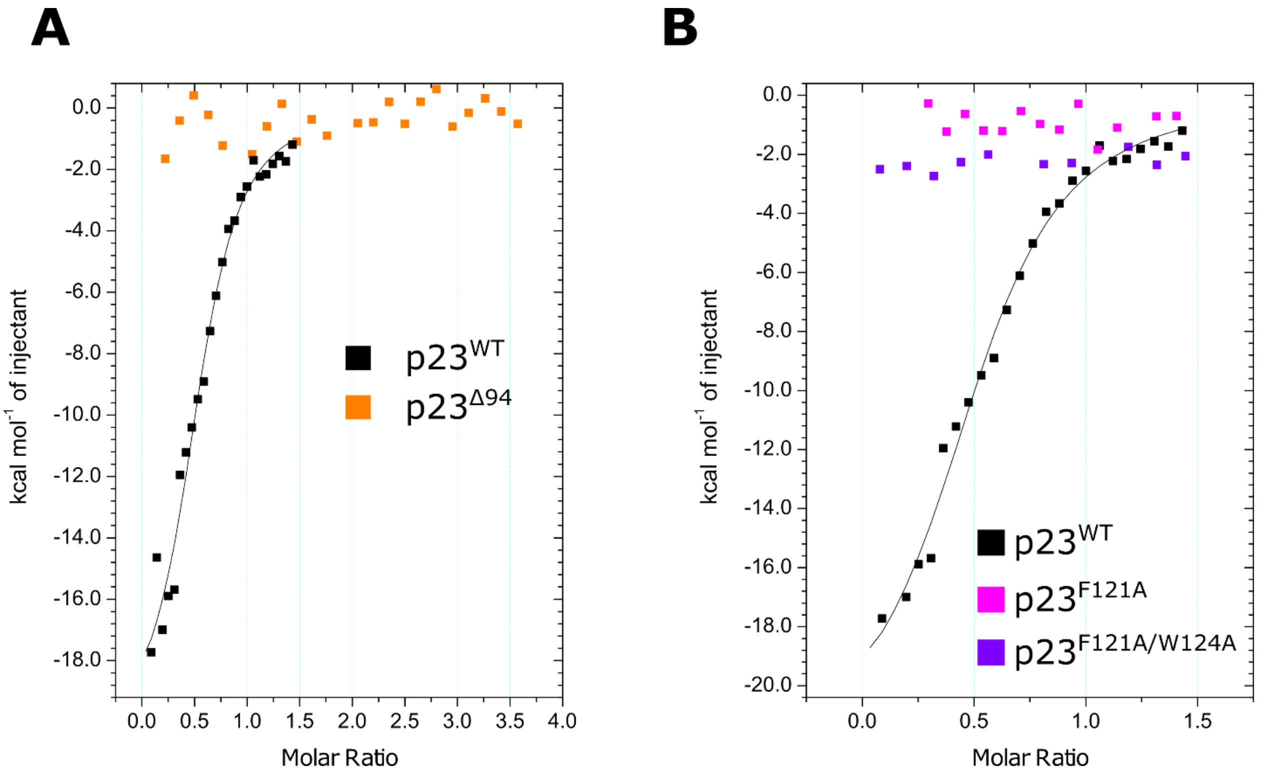

Supplementary Figure 1: Isothermal Titration Calorimetry of p23 mutants

(A) + **(B)** The binding of p23WT and the depicted p23 mutants to Hsp90 was tested using isothermal titration calorimetry (ITC). While the WT mutant readily binds to Hsp90, none of the mutants interacts with Hsp90.

Supplementary Figure 2

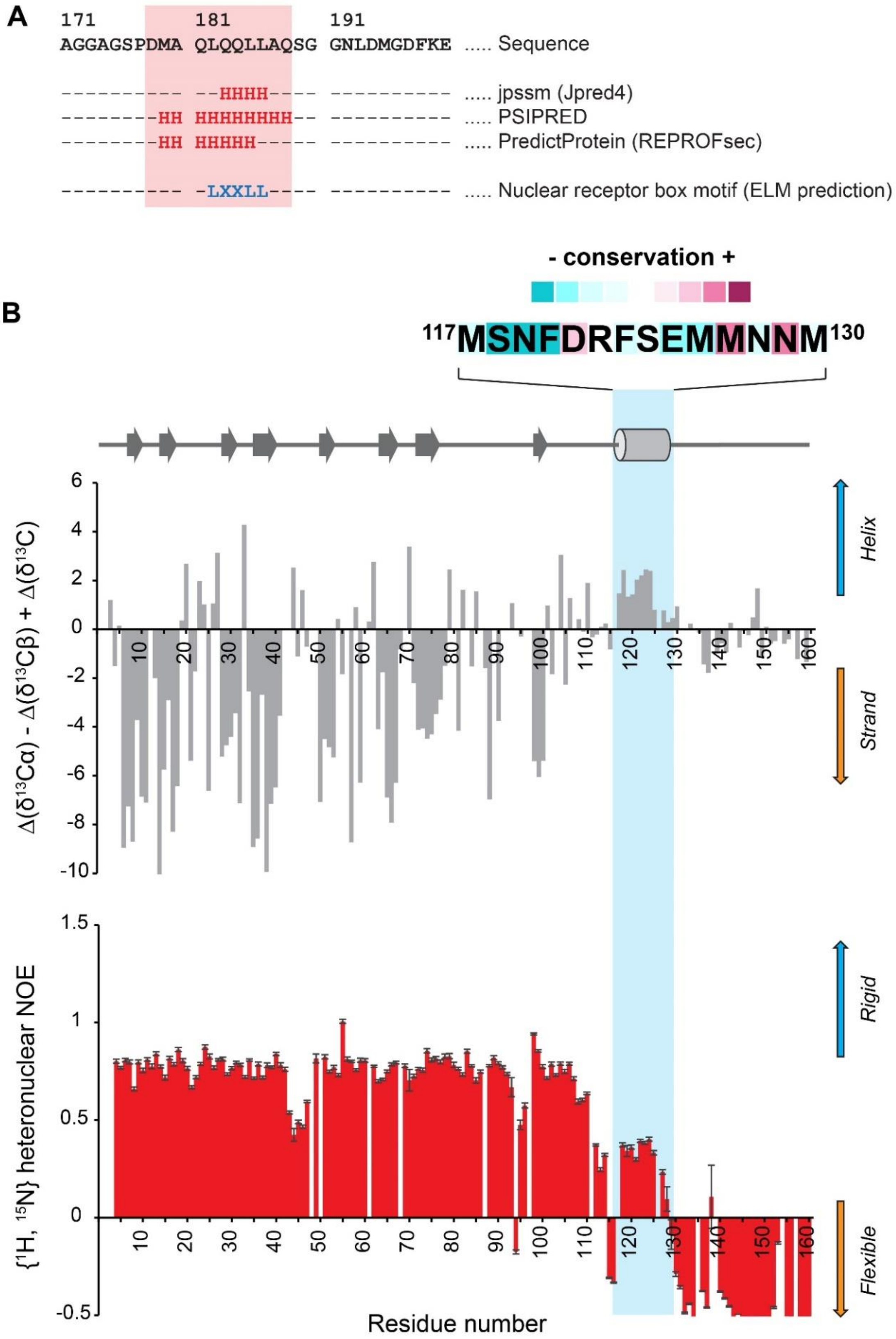

Supplementary Figure 2: Yeast and human p23 harbor a structured element in the tail

(A) The shown prediction algorithms were used to identify potential secondary structures in the yeast p23 tail. The sequence also contains a LxxLL motif for steroid hormone receptor binding, as determined by ELM resource [1].

(B) Secondary ^{13}C chemical shifts of human p23 using previously published data set from Dyson and coworkers [2], complemented with $^{13}C'$ data. Also in human p23, a helical segment is predicted containing HXXHH motif (^{123}FSEMM^{127}, H = hydrophobic amino acid). Amino acid conservation obtained from Consurf analysis [3] is indicated at the top according to the color scale. $\{^1H\}$-^{15}N heteronuclear NOE values obtained from the ratio of peak intensities of saturated *vs.* non-saturated experiments. Errors were estimated from spectral baseplane noise RMSD according to Farrow et al. [4].

Supplementary Figure 3

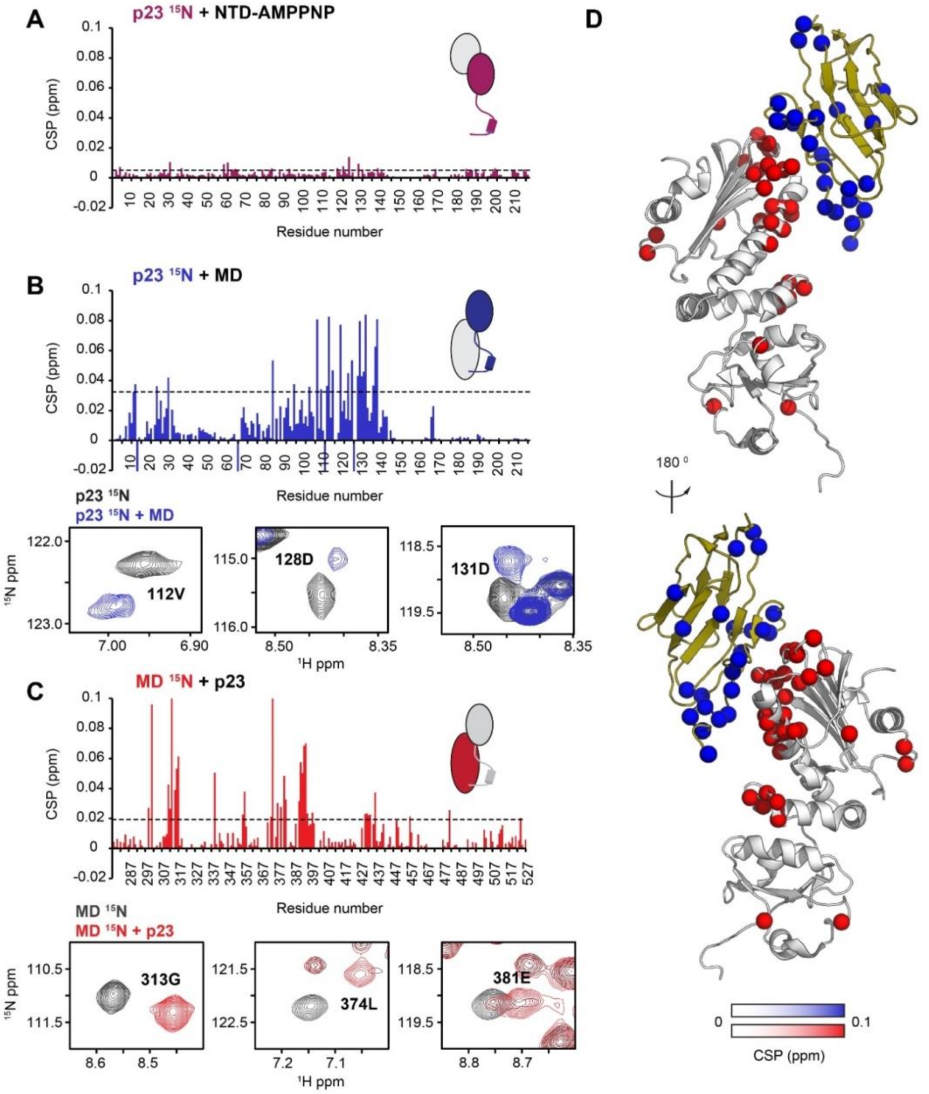

Supplementary Figure 3: NMR analysis of the binding between p23 and individual domains of Hsp90

(A) CSP plot of ^{15}N labeled p23 upon binding of NTD-AMPPNP. The absence of significant changes indicate no interaction. In A-C, median + 1σ is shown as a dotted line.

(B) CSP plot of ^{15}N labeled p23 upon binding of MD. Zoomed views of representative peaks are shown in the bottom; spectra of the non-complexed and complexed forms are shown in gray and blue, respectively.

(C) CSP plot of ^{15}N labeled MD upon binding of p23. Zoomed views of representative peaks are shown in the bottom; spectra of the non-complexed and complexed forms are shown in gray and red, respectively.

(D) CSP plotted on the Hsp90 MD-p23 complex derived from the FL crystal structure, with larger chemical shifts depicted as red (Hsp90) and blue (Sba1) spheres. MD is colored in white and p23 in gold.

Supplementary Figure 4

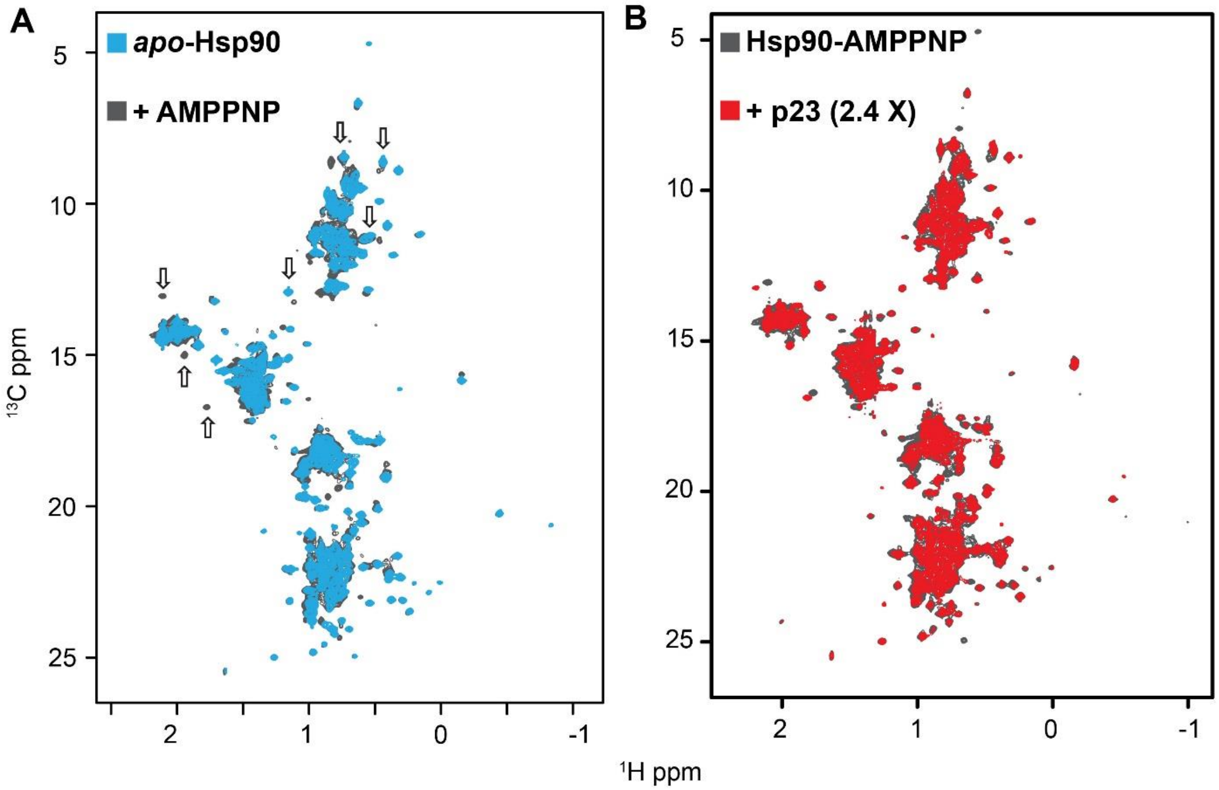

Supplementary Figure 4: ^{1}H-^{13}C TROSY spectra of AIL^{pro-R}V^{pro-R}M labeled Hsp90

(A) Spectra comparison of *apo*-Hsp90 (blue) and bound to AMPPNP (grey), indicating representative views of second set of resonances corresponding to the closed conformation (arrows).

(B) Spectrum of Hsp90-AMPPNP in complex with p23 (red) overlay with the non-complexed spectrum of Hsp90-AMPPNP (gray).

Supplementary Figure 5

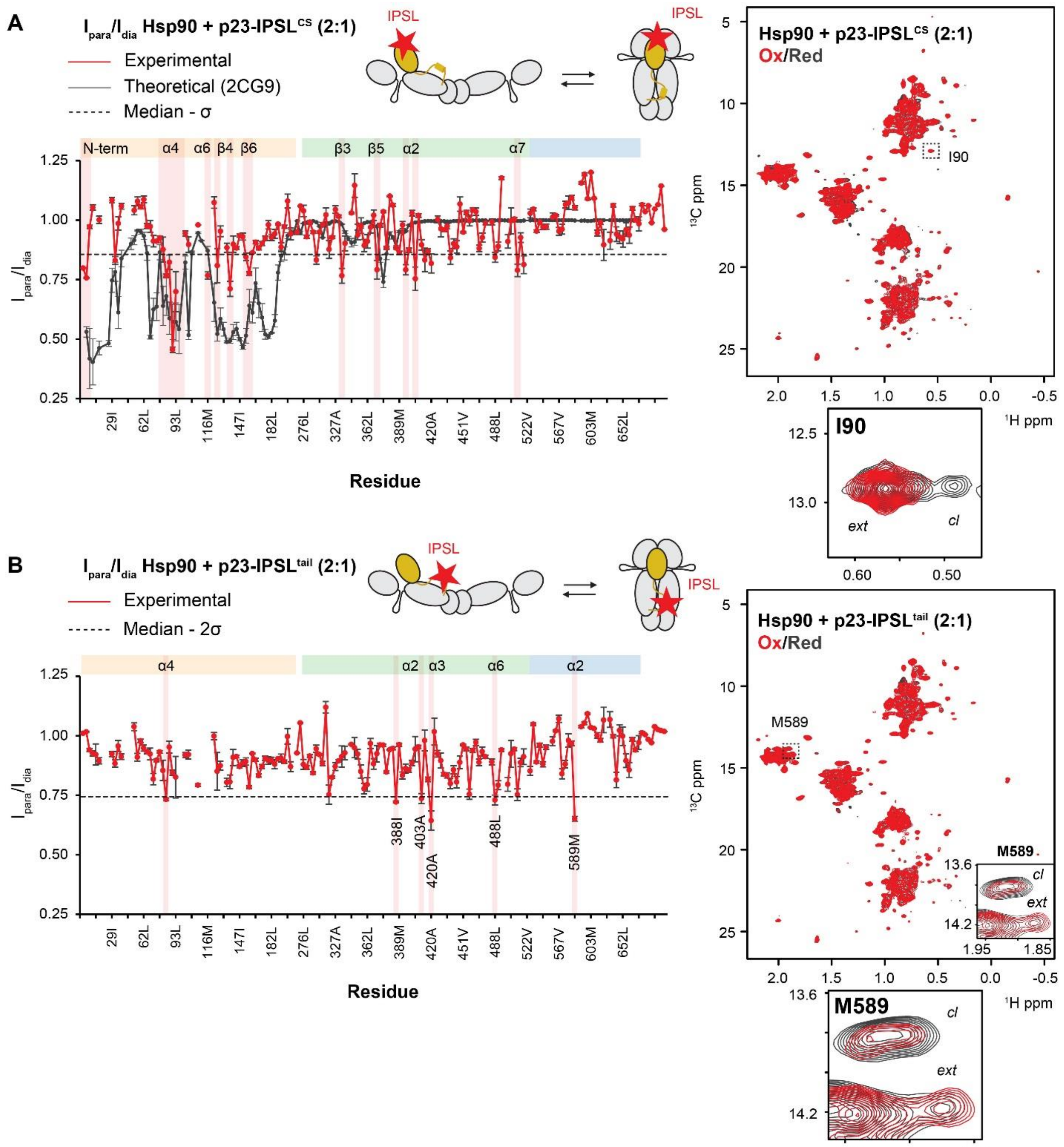

Supplementary Figure 5: Intermolecular PRE experiments with p23 spin-labeled in the CS core domain or the tail

(A) Intensity ratios between oxidized and reduced states of methyl labeled Hsp90 in complex with p23 spin labeled at residue Cys35 (CS domain) *vs.* residue number, in red. Overlaid spectra of the oxidized (red) and reduced (black) forms are included at the right. An example of conformation-specific PRE is shown below. Experiments were performed at 100 µM of Hsp90

and p23 spin labeled at 2:1 ratio, which correspond to 80% of a 2:1 complex of Hsp90:p23, according to the stoichiometry obtained by ITC. Median - 1σ is shown as a dotted line. Theoretical intensity ratios derived from the crystal structure of the complex (PDB: 2CG9) are shown in gray. Only one paramagnetic center was used for the calculations by artificially removing one p23 chain. In order to account for the different paramagnetic effects on the two protomers, the intensity ratios for protomers A and B were averaged. Ratios were obtained as described previously [5]. In A and B, residues showing larger paramagnetic effects are highlighted in red and indicated at the top. Reported data correspond to the closed conformation signals for those residues showing two sets of signals.

(B) Intensity ratio plot for the complex of Hsp90 with p23 mutant C35A/S189C spin labeled at the C-tail helix, recorded in the same conditions as in **(A)**. Median - 2σ is shown as a dotted line. Overlaid spectra of the oxidized (red) and reduced (black) forms are included at the right, with an example of conformation-specific PRE shown in the inset

Supplementary Figure 6

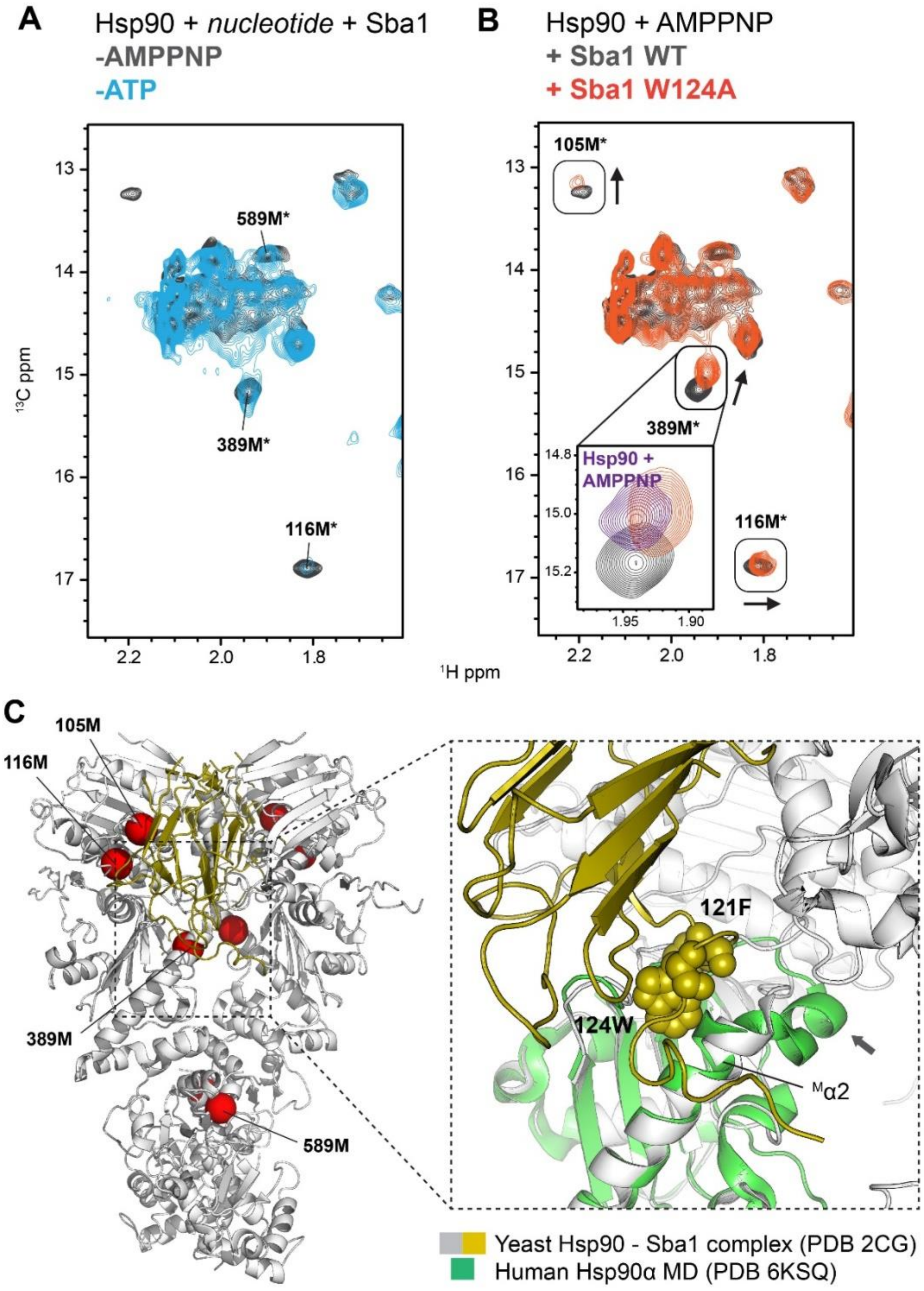

Supplementary Figure 6: Effects of p23 on the closed conformation of Hsp90

(A) Nucleotide effects on the spectra of Hsp90 in complex with p23. Zoomed view of the Met region of ^{1}H-^{13}C TROSY spectra of methyl labeled Hsp90 bound to AMP-PNP (grey) and to ATP (blue). The high similarity on the signals corresponding to the closed state (marked with asterisks) of Hsp90:p23 in the two cases indicate that p23 triggers similar changes to the closed conformation independent of the nucleotide type

(B) W124A mutant results in impaired conformational switching. The magnified view of the Met region of the spectra of Hsp90-AMP-PNP bound to 1.2 molar excess of Sba1 WT (grey) and to the inhibitory defective mutant W124A (orange) shows slight shifts on the peaks corresponding to the closed conformation (marked with asterisks). Particularly, the signal of M389 in the closed state resembles the non-complexed state (purple), indicating that this mutant is unable to trigger shifts on the N-terminus of α2 of the MD involved in catalysis.

(C) Residues indicated in A) and B) plotted in the crystal structure of Hsp90:p23 complex as red spheres (PDB: 2CG9). A zoomed view of the N-terminus of α2 of the MD is shown at the right, with the crystal structure of the MD from human Hsp90α aligned in green (PDB: 6KSQ). Differences on the conformation are indicated by an arrow.

Supplementary Figure 7

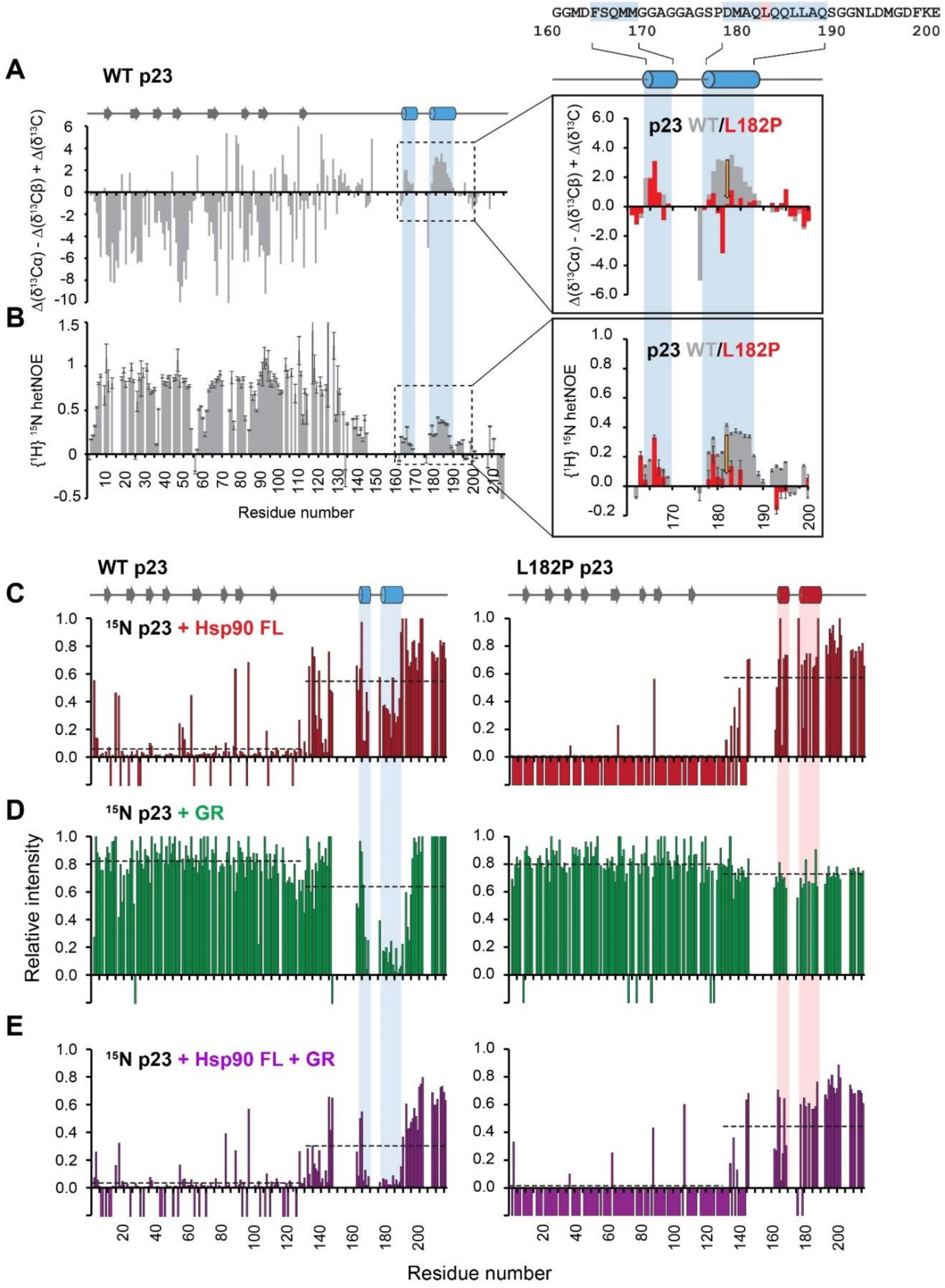

Supplementary Figure 7: L182P mutation in C-tail helix of p23 shows that a helical motif is required for interactions with Hsp90 bound to AMP-PNP, and/or the glucocorticoid receptor ligand binding domain.

(A) Comparison of the ^{13}C secondary chemical shifts of p23^{L182P} (red) with those of p23^{WT} (gray) shows that this substitution disrupts the helix. {^{1}H}-^{15}N heteronuclear NOE values obtained from the ratio of peak intensities of saturated *vs.* non-saturated experiments. Errors were estimated from spectral baseplane noise RMSD according to Farrow et al. [4]. Low hetNOE values of the mutant are also consistent with this notion. **(B)** The position of the mutation is marked by an arrow **(C-E)** Relative peak intensities of p23^{WT} (left) and p23^{L182P} (right) in the absence and the presence of **(C)** Hsp90 FL, **(D)** GR-LBDm, or **(E)** Hsp90 FL and GR-LBDm. Secondary structure elements are shown at the top, highlighting the tail fragment showing helical propensity.

Supplementary References

1 Kumar, M. *et al.* ELM—the eukaryotic linear motif resource in 2020. *Nucleic Acids Research* **48**, D296-D306, doi:10.1093/nar/gkz1030 (2020).

2 Martinez-Yamout, M. A. *et al.* Localization of sites of interaction between p23 and Hsp90 in solution. *J. Biol. Chem.* **281**, 14457-14464, doi:10.1074/jbc.M601759200 (2006).

3 Glaser, F. *et al.* ConSurf: Identification of functional regions in proteins by surface-mapping of phylogenetic information. *Bioinformatics* **19**, 163-164, doi:10.1093/bioinformatics/19.1.163 (2003).

4 Farrow, N. A. *et al.* Backbone dynamics of a free and phosphopeptide-complexed Src homology 2 domain studied by 15N NMR relaxation. *Biochemistry* **33**, 5984-6003, doi:10.1021/bi00185a040 (1994).

5 Simon, B., Madl, T., Mackereth, C. D., Nilges, M. & Sattler, M. An efficient protocol for NMR-spectroscopy-based structure determination of protein complexes in solution. *Angewandte Chemie* **49**, 1967-1970, doi:10.1002/anie.200906147 (2010).
