# Supplementary material for: Structural elements in the flexible tail of the co-chaperone p23 coordinate client binding and progression of the Hsp90 chaperone cycle
Source: Nat Commun. 2021 Feb 5;12:828. doi: 10.1038/s41467-021-21063-0 (PMC7864943; doi:10.1038/s41467-021-21063-0)
Supplement: Supplementary file 2 — Reporting Summary [file 41467_2021_21063_MOESM2_ESM.pdf]

nature research

Corresponding author(s): Johannes Buchner, Michael Sattler

Last updated by author(s): Dec 21, 2020

Reporting Summary

Nature Research wishes to improve the reproducibility of the work that we publish. This form provides structure for consistency and transparency in reporting. For further information on Nature Research policies, see our Editorial Policies and the Editorial Policy Checklist.

Statistics

For all statistical analyses, confirm that the following items are present in the figure legend, table legend, main text, or Methods section.

n/a	Confirmed	
☐	☒	The exact sample size (*n*) for each experimental group/condition, given as a discrete number and unit of measurement
☐	☒	A statement on whether measurements were taken from distinct samples or whether the same sample was measured repeatedly
☐	☒	The statistical test(s) used AND whether they are one- or two-sided<br>*Only common tests should be described solely by name; describe more complex techniques in the Methods section.*
☒	☐	A description of all covariates tested
☒	☐	A description of any assumptions or corrections, such as tests of normality and adjustment for multiple comparisons
☐	☒	A full description of the statistical parameters including central tendency (e.g. means) or other basic estimates (e.g. regression coefficient) AND variation (e.g. standard deviation) or associated estimates of uncertainty (e.g. confidence intervals)
☐	☒	For null hypothesis testing, the test statistic (e.g. *F*, *t*, *r*) with confidence intervals, effect sizes, degrees of freedom and *P* value noted<br>*Give P values as exact values whenever suitable.*
☒	☐	For Bayesian analysis, information on the choice of priors and Markov chain Monte Carlo settings
☒	☐	For hierarchical and complex designs, identification of the appropriate level for tests and full reporting of outcomes
☒	☐	Estimates of effect sizes (e.g. Cohen's *d*, Pearson's *r*), indicating how they were calculated

Our web collection on statistics for biologists contains articles on many of the points above.

Software and code

Policy information about availability of computer code

Data collection	NMR spectra were collected using Bruker Topspin 3.5 software. Absorbance data was acquired using the Cary WinUV software.
Data analysis	Origin 2019, SEDVIEW, NMRPipe, CcpNmr 2.4.2

For manuscripts utilizing custom algorithms or software that are central to the research but not yet described in published literature, software must be made available to editors and reviewers. We strongly encourage code deposition in a community repository (e.g. GitHub). See the Nature Research guidelines for submitting code & software for further information.

Data

Policy information about availability of data

All manuscripts must include a data availability statement. This statement should provide the following information, where applicable:

- Accession codes, unique identifiers, or web links for publicly available datasets
- A list of figures that have associated raw data
- A description of any restrictions on data availability

The NMR assignments of methyl-labeled yeast Hsp90 have been deposited to the Biological Magnetic Resonance Bank (BMRB) under accession code 50474.

Field-specific reporting

Life sciences study design

All studies must disclose on these points even when the disclosure is negative.

Sample size	In NMR experiments, a single experiment was performed. For the remaining biophysical experiments, the sample size is stated in the figure legends.
Data exclusions	No data were excluded from the analyses.
Replication	All data presented in the manuscript are reproducible. The number of replicates are stated in the figure legends and replicates were performed independently.
Randomization	Randomization is not possible in biochemical research. For cellular studies in yeast, biological replicates were performed: Random colonies were used for each measurement. For biochemical experiments, independent reactions were measured.
Blinding	Not applicable. Blinding is not possible in biochemical research: The concealment of group allocation is not required and not possible.

Reporting for specific materials, systems and methods

We require information from authors about some types of materials, experimental systems and methods used in many studies. Here, indicate whether each material, system or method listed is relevant to your study. If you are not sure if a list item applies to your research, read the appropriate section before selecting a response.

Materials & experimental systems

n/a	Involved in the study	
☒	☐	Antibodies
☒	☐	Eukaryotic cell lines
☒	☐	Palaeontology and archaeology
☒	☐	Animals and other organisms
☒	☐	Human research participants
☒	☐	Clinical data
☒	☐	Dual use research of concern

Methods

n/a	Involved in the study	
☒	☐	ChIP-seq
☒	☐	Flow cytometry
☒	☐	MRI-based neuroimaging
